# Characterization of an NDM-5 carbapenemase-producing *Escherichia coli* ST156 isolate from a poultry farm in Zhejiang, China

**DOI:** 10.1186/s12866-019-1454-2

**Published:** 2019-04-25

**Authors:** Biao Tang, Jiang Chang, Liujie Cao, Qixia Luo, Hao Xu, Wentao Lyu, Mingrong Qian, Xiaofeng Ji, Qiaoyan Zhang, Xiaodong Xia, Hua Yang

**Affiliations:** 1 Institute of Quality and Standard for Agro-products, Zhejiang Academy of Agricultural Sciences, Hangzhou, 310021 Zhejiang China; 2 College of Food Science and Engineering, Northwest Agriculture and Forestry University, Yangling, 712100 Shaanxi China; 3 State Key Laboratory for Diagnosis and Treatment of Infectious Diseases, Collaborative Innovation Center for Diagnosis and Treatment of Infectious Diseases, The First Affiliated Hospital of Medicine School, Zhejiang University, Hangzhou, 310003 China; 4 State Key Laboratory Breeding Base for Zhejiang Sustainable Pest and Disease Control, Zhejiang Academy of Agricultural Sciences, Hangzhou, 310021 Zhejiang China

**Keywords:** *bla*_NDM-5_, Carbapenemase, *Escherichia coli*, Multidrug resistance, Poultry farm

## Abstract

**Background:**

The emergence of carbapenem-resistant *Enterobacteriaceae* strains has posed a severe threat to public health in recent years. The mobile elements carrying the New Delhi metallo-β-lactqtamase (NDM) gene have been regarded as the major mechanism leading to the rapid increase of carbapenem-resistant *Enterobacteriaceae* strains isolated from clinics and animals.

**Results:**

We describe an NDM-5-producing *Escherichia coli* strain, ECCRA-119 (sequence type 156 [ST156]), isolated from a poultry farm in Zhejiang, China. ECCRA-119 is a multidrug-resistant (MDR) isolate that exhibited resistance to 27 antimicrobial compounds, including imipenem and meropenem, as detected by antimicrobial susceptibility testing (AST). The complete genome sequence of the ECCRA-119 isolate was also obtained using the PacBio RS II platform. Eleven acquired resistance genes were identified in the chromosome; four were detected in plasmid pTB201, while six were detected in plasmid pTB202. Importantly, the carbapenem-resistant gene *bla*_NDM-5_ was detected in the IncX3 plasmid pTB203. In addition, seven virulence genes and one metal-resistance gene were also detected. The results of conjugation experiments and the transfer regions identification indicated that the *bla*_NDM-5_-harboring plasmid pTB203 could be transferred between *E. coli* strains.

**Conclusions:**

The results reflected the severe bacterial resistance in a poultry farm in Zhejiang province and increased our understanding of the presence and transmission of the *bla*_NDM-5_ gene.

**Electronic supplementary material:**

The online version of this article (10.1186/s12866-019-1454-2) contains supplementary material, which is available to authorized users.

The overuse of antibiotics has led to the emergence of a large number of multidrug-resistant pathogens, which constitutes a serious threat to public health [1]. Imipenem and meropenem are carbapenem antibiotics that have been used as last resorts in the treatment of infections caused by gram-negative bacteria, especially multidrug-resistant gram-negative pathogens [2]. In 2008, a novel carbapenem resistance gene, New Delhi metallo-β-lactamase (NDM), was detected in *Klebsiella pneumoniae* isolated from a Swedish patient of Indian origin. This gene attracted international attention for the high level of resistance it confers to bacteria against most β-lactams, except aztreonam, and its spread to over 50 countries [3]. The NDM variant NDM-5 was first reported in 2011 in *Escherichia coli* isolated from a patient in the United Kingdom who had received treatment in India [4]. Subsequently, NDM-5 was reported in many other countries, including India [5], Algeria [6], Japan [7], South Korea [8], Australia [9], China [10], Denmark [11], Italy [12], America [13], Spain [14], Egypt [15], France [16], and New Zealand [17]. In China, many pathogens carrying *bla*_NDM-5_ have been isolated from patients [18–21]. In addition, *bla*_NDM-5_ can also be isolated from pigs [22, 23], dairy cows [24] and vegetables [25]. The complete sequences of *bla*_NDM-5_-harboring plasmids have been helpful for the study of the transmission of the *bla*_NDM-5_ gene, although not all of these plasmids have been reported.

In this study, we first describe the NDM-5-producing carbapenem-resistant *E. coli* strain, ECCRA-119, isolated from a layer hen farm in Zhejiang, China. We obtained the complete genome sequence, predicted the possible mechanism of multidrug resistance and assessed the transmission ability of the plasmid harboring *bla*_NDM-5_ from the ECCRA-119 isolate. These results increased our understanding of the diversity and complexity of the strains harboring *bla*_NDM-5_.

## Results

### Strain features

Two hundred nineteen of the samples studied tested positive for *E. coli*, and *E. coli* isolates from all of these samples were obtained and characterized by antimicrobial susceptibility testing (AST) using the VITEK® 2 COMPACT system (BioMérieux, France). The highest overall levels of resistance were observed toward ampicillin, with 74.43% of all isolates resistant to this antimicrobial. High rates of resistance were also observed toward trimethoprim (54.34%), with lower levels of resistance observed toward piperacillin (1.83%), amikacin (2.29%), and amoxicillin (0.91%). No strain was determined to be resistant to tigecycline. One hundred eighty isolates (82.2%) were resistant to at least one antimicrobial agent, and 92 isolates (42.01%) were resistant to three or more antimicrobial agents. Of the 219 *E. coli* isolates, a carbapenem-resistant strain was identified that showed resistance toward ertapenem and imipenem, which is rare in poultry.

The minimum inhibitory concentrations (MICs) of the ECCRA-119 isolate toward different antibiotics are shown in Table 1. The ECCRA-119 isolate was susceptible to colistin (MIC < 0.125 mg/L), polymyxin B (MIC 1 mg/L) and amikacin (MIC ≤4 mg/L), exhibited intermediate resistance toward gentamicin (MIC 8 mg/L), and was resistant to 27 different compounds from 7 antimicrobial classes that are frequently used in medical treatments, food animal feed and animal medicine (Table 1). In particular, this isolate was resistant to two carbapenems, imipenem (MIC 4 mg/L) and meropenem (MIC 8 mg/L). Therefore, we classified the ECCRA-119 isolate as a multidrug-resistant strain (MDR) due to its nonsusceptibility to many antimicrobial agents, including imipenem and meropenem.

**Table 1:** AST of the ECCRA-119 isolate using a panel of 46 antimicrobial agents

Antibiotic type		Antimicrobial Agent	MIC (mg/L)	R/I/S
β-lactams	Penicillins	Ampicillin^a^	> 512	R
		Ampicillin^b^	> 64	R
		Ampicillin/Sulbactam^b^	> 64/32	R
		Amoxicillin/ClavulaniC Acid^a^	128/64	R
		Amoxicillin/Clavulanate^b^	> 64/32	R
	Cephalosporins	Ceftiofur^a^	256	R
		Ceftazidime^a^	> 256	R
		Cefazolin^b^	> 16	R
		Cefoxitin^b^	> 64	R
		Cefotaxime^b^	> 8	R
		Ceftazidime^b^	> 16	R
		Cefepime^b^	> 16	R
		Cefotaxime/Clavulanate^b,c^	> 4/4	/
		Ceftazidime/Clavulanate^b,c^	> 8/4	/
	Monobactams	Aztreonam^b^	> 32	R
Carbapenems		Meropenem^a^	8	R
		Meropenem^b^	> 4	R
		Imipenem^b^	4	R
Tetracycline		Tetracycline^a^	128	R
		Tetracycline^b^	> 32	R
		Minocycline^b^	16	R
		Doxycycline^a^	32	R
		Doxycycline^b,c^	16	/
Aminoglycosides		Gentamicin^a^	8	I
		Gentamicin^b^	16	R
		Amikacin^b^	≤4	S
		Kanamycin^b^	> 64	R
		Streptomycin^b,c^	16	/
		Spectinomycin^a^	256	R
Sulfonamides		Sulfisoxazole^a^	> 512	R
		Sulfisoxazole^b,c^	> 512	/
		Trimethoprim/Sulfamethoxazole^a^	> 32/608	R
		Trimethoprim/Sulfamethoxazole^b^	> 8/152	R
Amphenicols		Florfenicol^a^	256	R
		Chloramphenicol^b^	> 64	R
Fluoro-quinolones		Enrofloxacin^a^	> 32	R
		Ofloxacin^a^	64	R
		Levofloxacin^b^	> 8	R
		Ciprofloxacin^b^	> 32	R
		Gemifloxacin^b,c^	> 16	/
		Nalidixic^b^	> 64	R
Polymyxin		Colistin^a^	< 0.125	S
		Colistin^b^	< 0.5	S
		Polymycin B^b^	1	S
Mequindox		Mequindox^a,c^	16	/
Macrolides		Azithromycin^b,c^	64	/

^a^Livestock antibiotics;

^b^Medical antibiotics;

^c^No interpreted standard for this antibiotic

### Characterization of the genome sequence of strain ECCRA-119

**Fig. 1:**
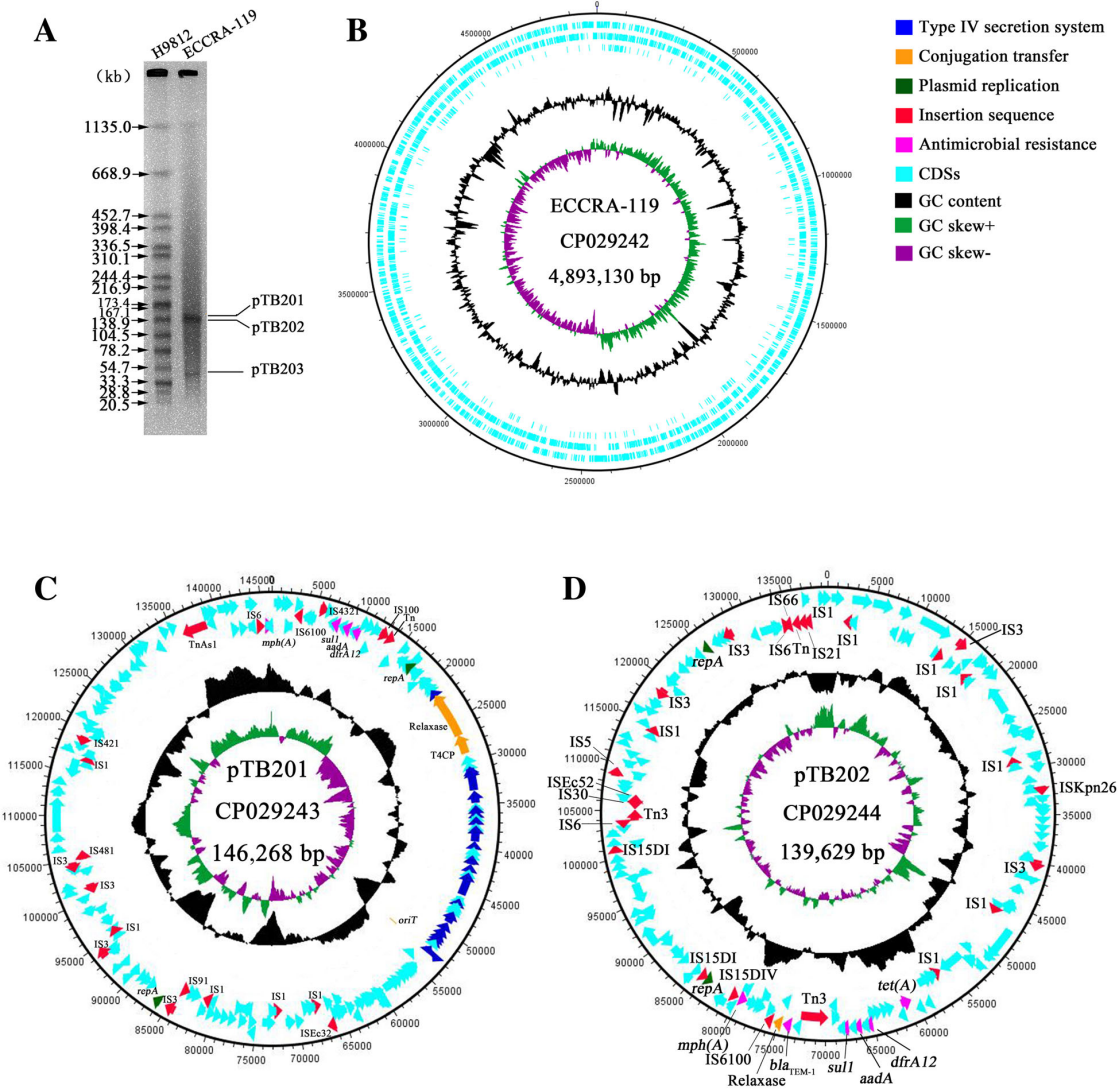
Representation of the completed chromosome and plasmids pTB201, pTB202 of the ECCRA-119 isolate. **a**: The S1-PFGE results of the ECCRA-119 isolate. **b**: The complete genome sequence map of the chromosome. **c**: The complete sequence map of plasmid pTB201. **d**: The complete sequence map of plasmid pTB202

**Fig. 2:**
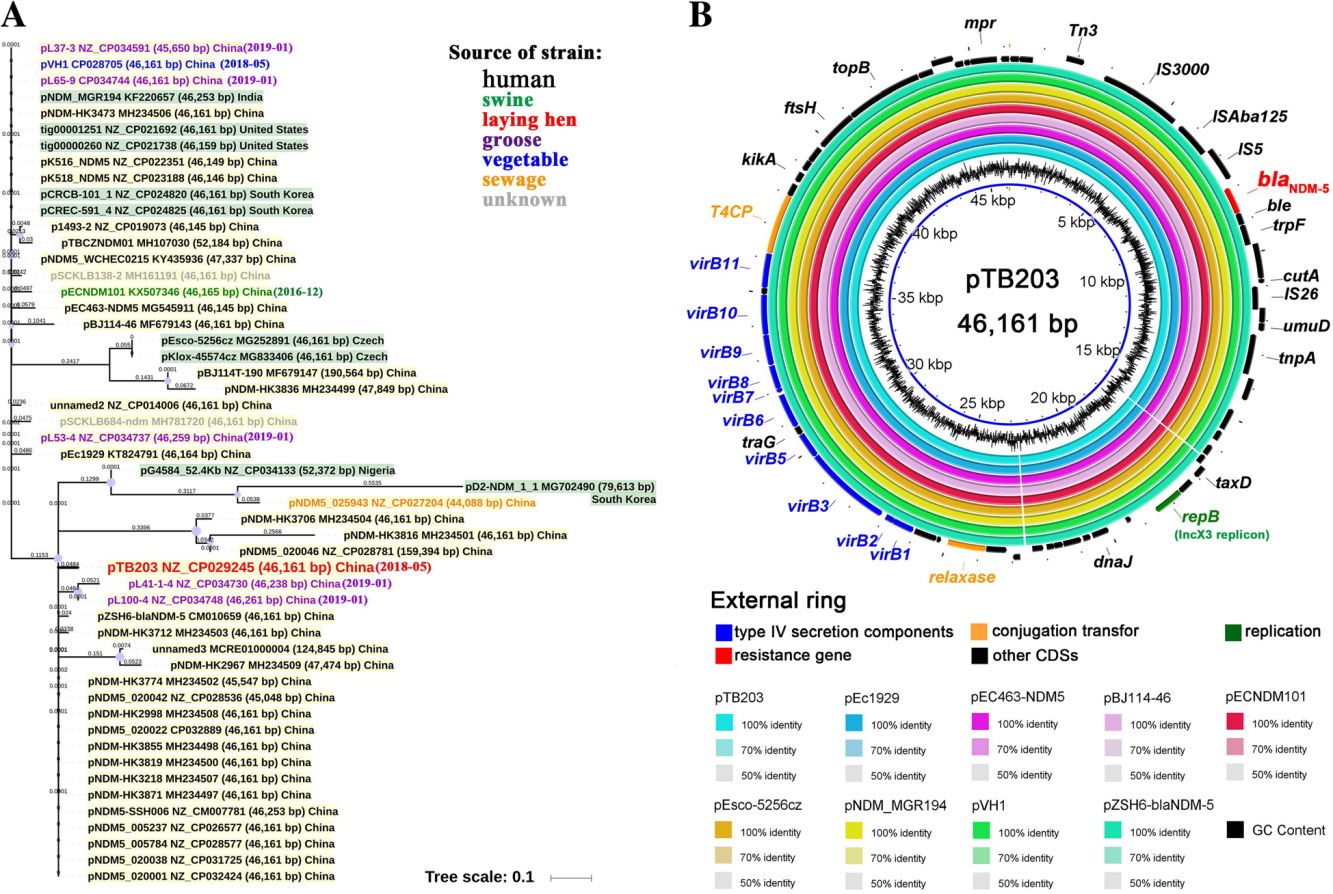
Phylogenetic and comparative analysis of IncX3 plasmids harboring *bla*_NDM-5_. **a**: The phylogenetic tree of 52 IncX3 plasmids from the GenBank database. Bar, 0.01 nucleotide substitutions per site. **b**: Comparative analysis among 9 published plasmids. The external ring represents the annotation of plasmid pTB203. Genes are color-coded depending on functional annotations

The genome of the ECCRA-119 isolate consisted of a single circular chromosome and three circular plasmids (Table 2, Figs. 1 and 2b). The chromosome sequence of ECCRA-119 was determined to be 4,893,130 bp in length, have a GC content of 50.77% and encodes 5042 proteins that account for 90.96% of the genome. The average depth of coverage was 210.5×, and 22 rRNAs, 87 tRNAs, and 2 CRISPRs were detected. Three plasmids in the ECCRA-119 isolate were identified, pTB201, pTB202 and pTB203. The plasmid pTB201, which is a combination of IncFII- and IncFIB-type plasmid, was determined to be 146,268 bp in length and have an average GC content of 51.35%. The plasmid pTB202, a p0111-IncN-type plasmid, was determined to be 139,629 bp in length and have an average GC content of 49.13%. In addition, the *bla*_NDM-5_-harboring plasmid pTB203, an IncX3-type plasmid, was determined to be 46,161 bp in length and have an average GC content of 46.65%. Moreover, the three plasmids were characterized by S1-PFGE (Fig. 1a), the results of which were consistent with the whole genome sequencing analysis. Multilocus sequence typing (MLST) analysis classified *E. coli* ECCRA-119 as ST156, suggesting that *E. coli* ST156 strains have the potential to harbor *bla*_NDM-5_-like genes.

**Table 2:** Characteristic features of the genome of the ECCRA-119 isolate

Features	ECCRA-119			
GenBank	CP029242	CP029243	CP029244	CP029245
Status	Chromosome	Plasmid	Plasmid	Plasmid
Genome size (bp)	4,893,130	146,268	139,629	46,161
G + C content (%)	50.77	51.35	49.13	46.65
No. of predicted coding sequences (CDS)	5042	199	170	64
rRNA	22	0	0	0
tRNA	87	0	0	0
No. of CRISPR regions	2

Twenty-two acquired resistance genes were identified in the ECCRA-119 genome that belong to eight antibiotic resistance categories (Table 3). Among these genes, 11 are located on the chromosome, four on plasmid pTB201, six on plasmid pTB202 and one on plasmid pTB203. In addition, several gene mutations were identified in the quinolone and fluoroquinolone resistance-determining region on the chromosome (Additional file 1: Table S1). Double *gyrA* mutations (giving rise to the amino acid substitutions S83 L and D87Y), *parC* mutation (giving rise to the amino acid substitution S80I) and *parE* mutations (giving rise to the amino acid substitution S458A) were also predicted in the ECRRA-119 isolate.

**Table 3:** Acquired antibiotic resistance genes of strain ECCRA-119

Resistance gene	Location	Position in contig	Phenotype
*aph(4)-Ia*	chromosome	74,566..75,591	Aminoglycoside resistance
*aac(3)-IVa*	chromosome	75,812..76,596	Aminoglycoside resistance
*aadA2*	chromosome	77,467..78,055	Aminoglycoside resistance
*aph(3′)-Ia*	chromosome	80,259..81,074	Aminoglycoside resistance
*bla*_CTX-M-65_	chromosome	84,756..85,631	Beta-lactam resistance
*fosA3*	chromosome	55,199..55,615	Fosfomycin resistance
*mdf(A)*	chromosome	3,166,104..3,167,336	MLS resistance
*floR*	chromosome	65,187..66,400	Phenicol resistance
*sul2*	chromosome	68,904..69,719	Sulphonamide resistance
*tet(B)*	chromosome	4,226,971..4,228,176	Tetracycline resistance
*dfrA12*	chromosome	78,463..78,960	Trimethoprim resistance
*aadA2*	pTB201	8,213..9,004	Aminoglycoside resistance
*mph(A)*	pTB201	145,592..146,268	Macrolide resistance
*sul1*	pTB201	6,859..7,708	Sulphonamide resistance
*dfrA12*	pTB201	9,412..9,909	Trimethoprim resistance
*aadA2*	pTB202	66,374..67,135	Aminoglycoside resistance
*bla*_TEM-1B_	pTB202	73,633..74,493	Beta-lactam resistance
*mph(A)*	pTB202	78,775..79,680	Macrolide resistance
*sul1*	pTB202	67,670..68,509	Sulphonamide resistance
*tet(A)*	pTB202	60,435..61,634	Tetracycline resistance
*dfrA12*	pTB202	65,469..65,966	Trimethoprim resistance
*bla*_NDM-5_	pTB203	7,921..8,733	Beta-lactam resistance

Seven virulence factors were detected in the whole genome sequence (Additional file 1: Table S2), four in the chromosome and three in the pTB201 plasmid, indicating the potential virulence of the ECCRA-119 isolate. These virulence factors are grouped into five classes (*iss*, *gad*, *lpfA*, *iroN*, and *cma*), which are related to serum survival, glutamate decarboxylase, long polar fimbriae, enterobactin siderophore receptor protein, and colicin M, respectively. In addition, one mercury resistance-related gene, *merA*, was identified on plasmid pTB201 (Additional file 1: Table S3).

### Transferability of plasmids

Conjugation assays confirmed that *bla*_NDM-5_ could be transferred between *E. coli* strains, with an observed transfer frequency of (1.39 ± 0.12) × 10^− 5^. The antibiotic susceptibility testing results showed that the transconjugants, confirmed by PCR and sequencing, were resistant to meropenem (4 mg/L). The transfer regions of the three plasmids of strain ECCRA-119 were successfully identified (Figs. 1 and 2b) by oriTfinder, including the origin of transfer region (*oriT*), relaxase gene, bacterial type IV secretion system (T4SS) apparatus gene clusters and the type IV coupling protein (T4CP) gene. Plasmid pTB201 was observed to possess an *oriT* (52,884–52,969 bp in the plasmid), relaxase gene, T4CP and T4SSs, indicating a high potential for self-transferability [26]. Plasmid pTB202 was observed to harbor a relaxase but lacked an *oriT*, T4CP and/or T4SS, indicating that it is not a mobilizable plasmid [26]. Plasmid pTB203 possess a relaxase gene, T4CP and T4SSs, but lacked a typical *oriT* sequence, demonstrating its potential to be transferred to other bacteria [26], with its transfer ability having been confirmed experimentally.

### Phylogenetic analysis of strain ECCRA-119 with other *E. coli* ST156 isolates

MLST analysis classified *E. coli* strain ECCRA-119 as ST156. Thus, we built a phylogenetic tree to determine its relationship among ST156 *E. coli* strains based on a SNP analysis (Additional file 1: Figure S1). We identified 52,076 SNPs from the 37 genome sequences available in GenBank. Of these, 17,953 and 34,123 were identified as core and noncore SNPs, respectively. We excluded the noncore SNPs for the further analysis and constructed a phylogenetic tree based on the genome-wide core SNPs. The core genome analysis identified 5 groups (Additional file 1: Figure S1). *E. coli* strain ECCRA-119 is grouped with the strains 174,900, SCEC020022 and VREC0575, which were isolated from Bangladesh, China and the United Kingdom, respectively. There were 7 group-specific core SNPs in this group. The number of strain-specific SNPs identified in strains ECCRA-119, 174,900, SCEC020022 and VREC0575 was 59, 71, 134 and 160, respectively. Interestingly, most isolates identified from the same region or source are not in the same lineage. Isolates from different countries were observed to be clustered together (strains 157–1949 and SE11). Similarly, strains isolated from different hosts (wild animals, livestock and poultry, and dog) clustered into the same branch (strains MOD1-EC5693, CVM N33633PS and MOD1-EC6498).

### Phylogenetic and comparative analysis of pTB203 and other *bla*_NDM-5_-harboring IncX3 plasmids

An SNP-based phylogenetic analysis was conducted using the 52 complete sequences of *bla*_NDM-5_-harboring IncX3 plasmids available in GenBank (Fig. 2a). Among these sequences, 41 originated from bacterial strains from humans, 1 from a pig, 5 from geese, 1 from a vegetable, 1 from a layer hen, 1 from sewage, and 2 from unknown sources. Our results showed that the IncX3 plasmids have an extensive host range. Among these 52 plasmids, 43 were isolated in China and 33 were from *E. coli*. Five plasmids from geese became available in January 2019 but were not published. Among these plasmids, 9 published plasmids were selected and constructed by BRIG (Fig. 2b), including pVH1 (vegetable, China, 46,161 bp) [25], pNDM_MGR194 (human, India, 46,253 bp) [27], pECNDM101 (swine, China, 46,165 bp) [23], pEC463-NDM5 (human, China, 46,145 bp) [28], pBJ114–46 (human, China, 46,161 bp) [29], pEsco-5256cz (human, Czech, 46,161 bp) [30], pEc1929 (human, China, 46,164 bp) [31], pTB203 (layer hen, China, 46,161 bp, in this study), and pZSH6-blaNDM-5 (human, China, 46,161 bp) [32]. The results of BLAST homology analyses showed that these plasmids had more than 99.9% identity and 99.8% query coverage with each other. The comparative analysis of 9 *bla*_NDM-5_-harboring IncX3 plasmids (~ 46 kb) revealed that these plasmids are highly similar to each other, possessing the same backbone that includes the IncX3 replication, *bla*_NDM-5_ gene and conjugation/type IV secretion components. This result was further confirmed by the comparative analysis of 52 *bla*_NDM-5_-harboring IncX3 plasmids, with the exception of pD2-NDM_1_1 (human, South Korea, 79,613 bp) (Additional file 1: Figure S2). The results of our analysis showed that *bla*_NDM-5_-harboring IncX3 plasmids with an ~ 46 kb backbone have extensive host adaptability in *Enterobacteriaceae*.

### Complete sequences of plasmids harboring *bla*_NDM_ variants from China

**Fig. 3:**
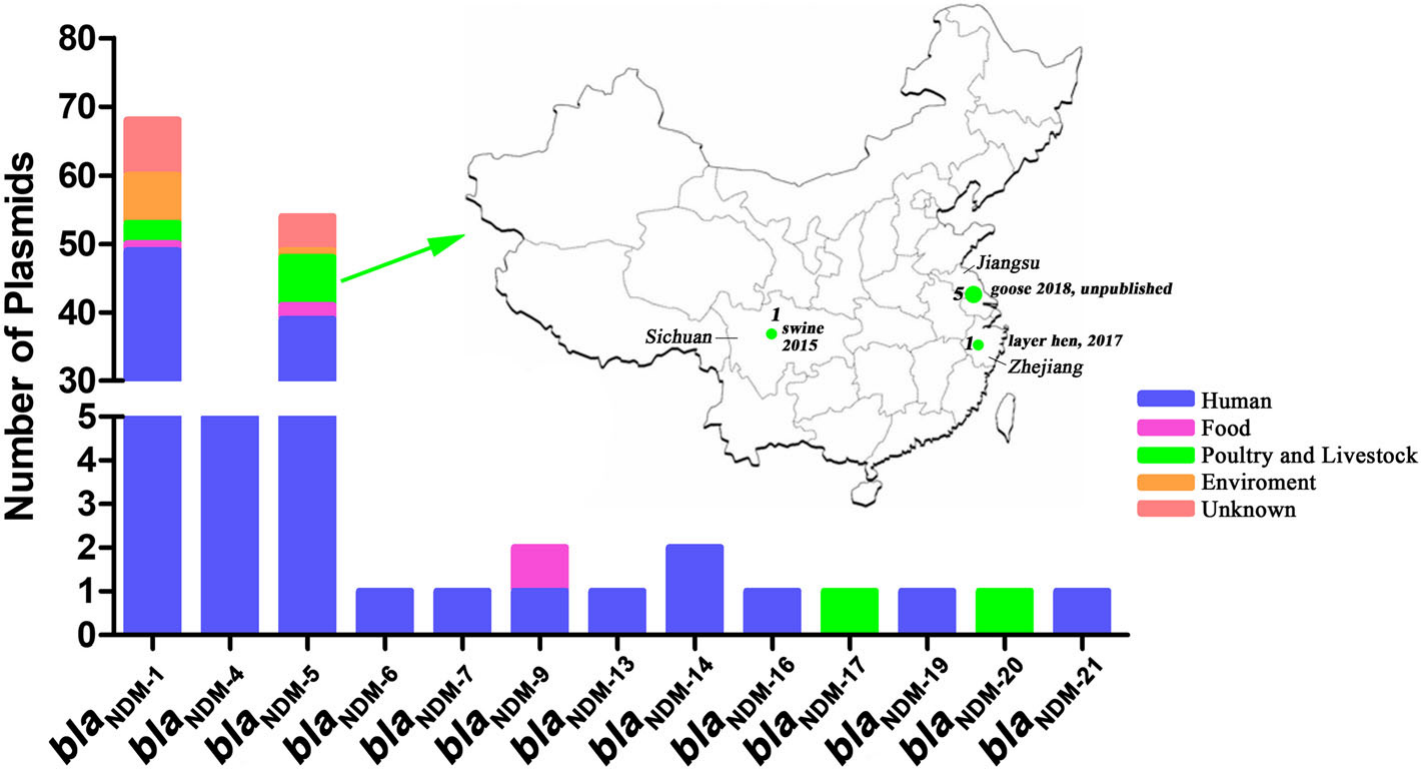
The distribution of *bla*_NDM_-harboring plasmids with complete sequences in China

At present, 24 variant *bla*_NDM_ sequences are available in GenBank, all of which were aligned by ClustalX (Additional file 1: Figure S3 and S4). These sequences are 813 bp in length, with the exception of *bla*_NDM-18_, and only 1–6 SNPs are observed among these sequences. In particular, the *bla*_NDM-5_ gene has the closest homology with *bla*_NDM-17_, *bla*_NDM-20_ and *bla*_NDM-21_ (Additional file 1: Figure S4). Relative to *bla*_NDM-5_, *bla*_NDM-17_, *bla*_NDM-20_ and *bla*_NDM-21_ contained point mutations at positions 508 (G → A), 809 (G → A), and 205 (G → A), generating amino acid substitutions Glu170Lys, Arg270His, and Gly69Ser, respectively. In China, 13 types of plasmids harbor *bla*_NDM_ genes with complete sequence are reported in GenBank, including *bla*_NDM-1_, *bla*_NDM-4_, *bla*_NDM-5_, *bla*_NDM-6_, *bla*_NDM-7_, *bla*_NDM-9_, *bla*_NDM-13_, *bla*_NDM-14_, *bla*_NDM-16_, *bla*_NDM-17_, *bla*_NDM-19_, *bla*_NDM-20_, *bla*_NDM-21_ (Additional file 2: Table S4, Fig. 3). The *bla*_NDM-1_ and *bla*_NDM-5_ genes are the most prevalent *bla*_NDM_ variants in China, with humans being the primary host source. Additionally, seven *bla*_NDM-5_-harboring plasmids have been detected in poultry and livestock in China, including 1 plasmid detected from swine in Sichuan in 2016, 1 plasmid detected from a layer hen in Zhejiang in 2017 (in this study), and 5 plasmids detected from geese in Jiangsu in 2018.

### Comparative analysis of plasmids pTB201 and pTB202

**Fig. 4:**
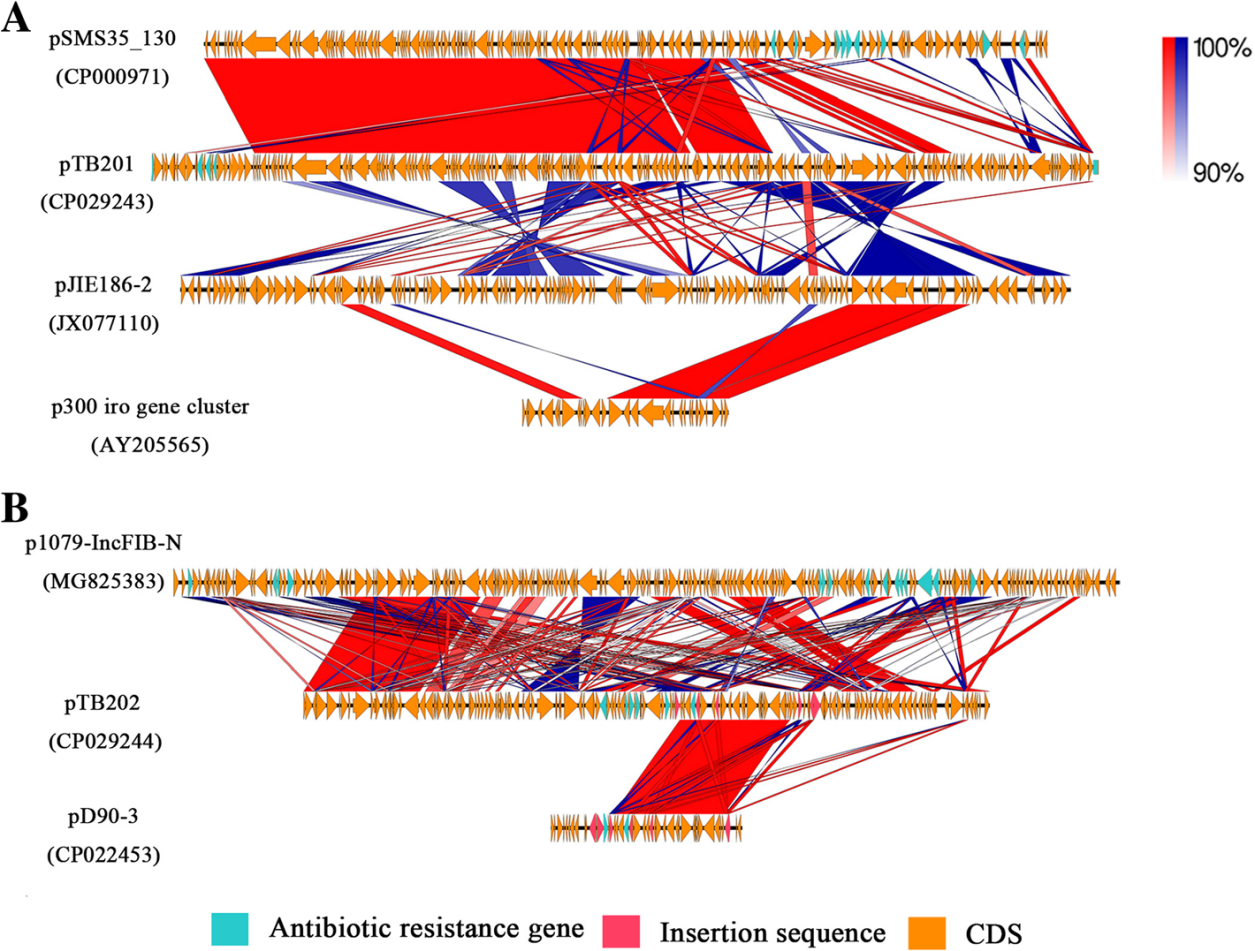
Comparative analysis of plasmids pTB201 and pTB202 with the corresponding homologous plasmids from GenBank. **a**: Comparative analysis of plasmid pTB201 (CP029243), pSMS35_130 (CP000971), pJIE186_2 (JX077110) and p300 iro gene cluster (AY205565). **b**: Comparative analysis of plasmid pTB202 (CP029244), p1079-IncFIB-N (MG825383) and pD90-3 (CP022453)

We compared the plasmids pTB201 and pTB202 with the corresponding homologous plasmids from GenBank via BLAST analyses. The results showed that plasmid pTB201 shares homology with plasmid pSMS35_130 (CP000971), plasmid pJIE186_2 (JX077110) and the p300 iro gene cluster (AY205565) (Fig. 4a); plasmid pTB202 showed homology with plasmid p1079-IncFIB-N (MG825383) and part of plasmid pD90–3 (CP022453) (Fig. 4b). These comparisons revealed that these plasmids do not have full-length matching plasmids in the GenBank database, although they shared backbones with many other plasmids.

## Discussion

The extensive use of antibacterials has led to the emergence of drug resistance as an increasingly serious issue, which poses a great threat to public health. There have been widespread reports of the isolation of multidrug resistant *E. coli* from hospitals, poultry, livestock, food, and the environment [33]. In this study, we identified a *bla*_NDM-5_-harboring *E. coli* isolate from a layer hen farm in Zhejiang, China, and we obtained detailed data through bioinformatics and experimental analyses. The AST results showed that the ECCRA-119 isolate is resistant to 27 different compounds used as therapeutics and food animal feeding, indicating its strong environmental adaptability under antibiotic selection pressure. There is no doubt that the multidrug resistance of this strain may present a serious risk to clinical and veterinary medicine. Apart from a few cases, acquired antimicrobial resistance genes and genomic mutations can largely explain drug resistance phenotypes. The identification of the antimicrobial resistance genes acquired by this strain show that this isolate may have a broad spectrum of drug resistance. For example, the presence of the *fosA3* gene in the chromosome could result in fosfomycin resistance [34], but further experiments are needed to confirm this possibility.

The *iss* gene was detected on both the chromosome and on plasmid pTB201, indicating the potential virulence of the ECCRA-119 isolate. The protein encoded by the *iss* gene is part of an outer membrane protein and is involved in the anti-complement effect of bacteria, possibly enhancing the serum resistance of *E. coli* and enabling the strain to rapidly proliferate in the host. It is widely believed that the *iss* gene is closely associated with the virulence of avian *E. coli* [35].

The *merA* gene was detected in plasmid pTB201, which can confer resistance to mercury and increase the viability of the ECCRA-119 isolate. Furthermore, the results suggested that plasmid pTB201 had a high potential for self-transferability. Therefore, it is likely that the mercury resistance of the ECCRA-119 isolate may be transferred to other bacteria [36]. Thus, the ECCRA-119 isolate has strong environmental resilience and a high potential to survival in a complicated breeding environment for a long time.

To the best of our knowledge, this is the first time a *bla*_NDM-5_-harboring plasmid has been reported in layer chickens. *E. coli* ST156 has not been a predominant multidrug-resistant clone observed worldwide in the past, but it is associated with the distribution of *bla*_NDM-1_ and *bla*_CTX-M-15_ in humans and poultry [37, 38]. The genes *mcr*-1 and *bla*_NDM-5_ have been reported to be detected in *E. coli* ST156 from Muscovy duck in China [39]. *E. coli* ST156 has spread to many countries and can be isolated from many types of hosts, suggesting that *E. coli* ST156 has the potential to play an important role in the transmission of the *bla*_NDM-5_ gene. In this study, the *bla*_NDM-5_-harboring plasmid was first detected from *E. coli* ST156 in the feces of a layer hen in China, which may increase our understanding of the transmission of *bla*_NDM-5_.

IncX3 plasmids are narrow host range plasmids of *Enterobacteriaceae* and are believed to have a low prevalence [40]. Since the first discovery of *bla*_NDM-5_ in China, this gene has been identified in a variety of *Enterobacteriaceae* [21, 31], with IncX3 being the primary type of Inc. to harbor *bla*_NDM-5_ [41]. From our results, the IncX3 plasmids harboring *bla*_NDM-5_ were highly similar to each other in different countries and host sources, suggesting its ability to be an efficient vehicle for *bla*_NDM-5_ dissemination among humans, animals, food and the environment, potentially indicating its role in the rapid spread of *bla*_NDM-5_-harboring isolates [21, 28]. The BRIG analysis results showed that *bla*_NDM-5_-harboring IncX3 plasmids have a conserved backbone of ~ 46 kb, indicating that these plasmids had a common ancestor, and the conjugation/type IV secretion components in the backbone may be a factor promoting its transmission.

The *bla*_NDM-5_-harboring plasmids were initially detected from isolates from human [4, 27, 28]. However, they have also been detected in food, the environment and livestock and poultry sources in recent years. For example, plasmid pNDM5_025943 (unpublished) was detected in sewage, and plasmid pVH1 was detected from a cucumber [25]. Carbapenem resistance is well known to be a universal phenomenon because of its frequent usage in clinics. Thus, it is interesting that the *bla*_NDM-5_-harboring plasmid has an increasing host range, which reflects the development of serious carbapenem resistance. In particular, the *bla*_NDM-5_ gene has been detected from livestock animals in recent years, such as swine [23] and dairy cows [24]. In this study, the complete sequence of a *bla*_NDM-5_-harboring plasmid isolated from layer hen feces was first published, which is important evidence of *bla*_NDM-5_ transmission in poultry in China.

## Materials and methods

### Sample collection and antimicrobial susceptibility testing

Using the sampling method proposed by Leon and Hassan [42, 43], 251 samples of chicken feces were collected from 12 large-scale chicken farms in Zhejiang province in 2017.

The *E. coli* isolate recovered was designated ECCRA-119 and showed resistance to meropenem (8 mg/L) and imipenem (4 mg/L). This isolate was selected for AST using the broth dilution method with the Biofosun® Gram-negative panel (Fosun Diagnostics, Shanghai, China). The criteria from the Clinical and Laboratory Standards Institute (CLSI) were used to interpret the results, and the US National Antimicrobial Resistance Monitoring System (NARMS) and European Committee on Antimicrobial Susceptibility Testing (EUCAST) protocol was used when CLSI standards were not appropriate. The panel of antimicrobial compounds tested included ampicillin, amoxicillin/clavulanic acid, tetracycline, doxycycline, gentamicin, spectinomycin, sulfisoxazole, trimethoprim/sulfamethoxazole, ceftiofur, ceftazidime, florfenicol, enrofloxacin, ofloxacin, colistin, meropenem, and mequindox from among livestock antibiotics, and ampicillin, ampicillin/sulbactam, tetracycline, chloramphenicol, trimethoprim/sulfamethoxazole, cefazolin, cefotaxime, ceftazidime, cefoxitin, gentamicin, imipenem, nalidixic acid, azithromycin, sulfisoxazole, ciprofloxacin, amoxicillin/clavulanate, Cefotaxime/clavulanate, ceftazidime/clavulanate, colistin, polymyxin B, minocycline, amikacin, aztreonam, cefepime, meropenem, levofloxacin, doxycycline, kanamycin, streptomycin, and gemifloxacin from among medical antibiotics.

### Whole genome sequencing, assembly and annotation

After genomic DNA extraction and quality checks, a 20-kb fragment library was constructed for the sample when the concentration and purity met the sequencing requirements. Whole-genome sequencing was performed using a PacBio RS II instrument [44]. The assembly of the reads was performed following the Hierarchical Genome Assembly Process (HGAP) workflow [45]. In this process, the Celera Assembler, following the OLC algorithm, was used to assemble the sequences [46], and Quiver was used to optimize the assembly results [45]. The gene prediction and annotation of the genomes was performed using the NCBI Prokaryotic Genome Annotation Pipeline [47]. The complete genome of the ECCRA-119 isolate was deposited in GenBank under the accession numbers CP029242 (chromosome), CP029243 (plasmid pTB201), CP029244 (plasmid pTB202) and CP029245 (plasmid pTB203).

### Sequence analysis

CRISPRfinder (https://crispr.i2bc.paris-saclay.fr/Server/) was used to search for CRISPR loci in the genome of the ECCRA-119 isolate [48]. MLST 2.0 (https://cge.cbs.dtu.dk/services/MLST/) was used to determine the ST [49]. The plasmid replicon types were identified using PlasmidFinder-1.3 (https://cge.cbs.dtu.dk/services/PlasmidFinder/) [50]. Acquired antimicrobial resistance genes were predicted using ResFinder (https://cge.cbs.dtu.dk/services/ResFinder/) [51]. VirulenceFinder (https://cge.cbs.dtu.dk/services/VirulenceFinder/) was used to identify the virulence factors [52], and oriTfinder (http://202.120.12.134/oriTfinder/oriTfinder.html) was used to identify the origin of transfer in the genome [53]. The genome was investigated for metal resistance genes using the Antibacterial Biocide and Metal Resistance Genes Database (BacMet) (http://bacmet.biomedicine.gu.se/) [54]. Easyfig [55] and BIRG [56] were used in the comparative analysis of the plasmids. Phylogenetic analysis of genome and plasmids was performed by KSNP based on the maximum-likelihood method [57]. Clustal X was used to perform the alignment analysis of *bla*_NDM_ based on nucleotide sequences [58]. The phylogenetic tree was generated used in MEGA X [59] and iTOL [60].

### Conjugation assay

Plasmid conjugation experiments were performed on the ECCRA-119 isolate as described previously by Lin et al. [23, 61]. A rifamycin-resistant *E. coli* EC600 strain was used as the recipient in the plasmid conjugation assay to test the transferability of the carbapenem resistance gene and other resistance genes harbored by the ECCRA-119 isolate. Briefly, transconjugants were selected on LB agar plates (Landbridge., Beijing, China) supplemented with rifamycin (400 mg/L) (Sangon Biotech., Shanghai, China) and meropenem (4 mg/L) (J&K Chemical Ltd., Shanghai, China). The transfer frequencies were calculated by dividing the number of colony-forming units (CFUs) of transconjugants by the number of CFUs of the recipients. Genome DNA was extracted from the *E. coli* transconjugant using a bacterial DNA extract kit (Generay, Shanghai, China). The *bla*_NDM-5_ primers (F: 5′-GTCTGGCAGCACACTTCCTA-3′; R: 5′- TAGTGCTCAGTGTCGGCATC-3′) were used to confirm that the transconjugant harbored the plasmid.

### S1-PFGE

S1-PFGE was performed according to a standard protocol using the contour-clamped homogeneous electric field (CHEF) technique with 0.5 × TBE buffer [62]. *Salmonella enterica* serotype Braenderup H9812 was used as a size marker [63]. The gels were run at 6 V/cm and 14 °C with an angle of 120°, and initial and final pulses were set at 2.16 and 63.8 s, respectively. The running time was 16 h using the CHEF apparatus (CHEF MAPPER XA; Bio-Rad, USA).

## Conclusions

In this study, we reported the isolation and characterization of a carbapenem-resistant *E. coli* strain ST156 harboring the *bla*_NDM-5_ gene from a layer hen farm in Zhejiang province, China. Three plasmids in ECCRA-119 were identified based on whole genome sequencing and S1-PFGE. Twenty-two acquired resistance genes were identified, and this finding is consistent with the MDR phenotype of strain ECCRA-119. In particular, the *bla*_NDM-5_ gene has a high risk of spreading widely due to the potential transfer ability of the IncX3 plasmid pTB203 in this strain. The results of our study may reflect the level of antimicrobial resistance in poultry breeding in Zhejiang province and increase our knowledge of the presence and transmission of the *bla*_NDM-5_ gene.

## Additional files

Additional file 1: **Table S1.** The gene mutation on the chromosome of strain ECCRA-119. **Table S2.** Virulence factors of strain ECCRA-119. **Table S3.** Metal resistance genes of strain ECCRA-119. **Figure S1.** SNP tree of *E. coli* ST156 strains. **Figure S2.** The comparison analysis of 52 *bla*_NDM-5_-harboring IncX3 plasmid sequences. **Figure S3.** Comparison analysis of 24 *bla*_NDM_ variant sequences based on nucleotide sequences. **Figure S4.** Phylogenetic relationships between *bla*_NDM_ variants based on nucleotide sequences. (DOCX 2669 kb)

Additional file 2: **Table S4.** The information for the *bla*_NDM_-harboring plasmids with complete sequences in China. (XLSX 20 kb)

## Abbreviations

AST: Antimicrobial susceptibility testing
BacMet: Antibacterial Biocide and Metal Resistance Genes Database
CFU: Colony-forming units
CHEFContour: clamped homogeneous electric field
CLSI: Clinical and Laboratory Standards Institute
EUCAST: European Committee on Antimicrobial Susceptibility Testing
HGAP: Hierarchical Genome Assembly Process
MDR: Multidrug resistant
MIC: Minimum inhibitory concentration
MLST: Multilocus sequence typing
NARMS: The US National Antimicrobial Resistance Monitoring System
NDM: New Delhi metallo-β-lactamase
*oriT*: Origin of transfer region
SNP: Single nucleotide polymorphism
T4CP: The type IV coupling protein
T4SS: Bacterial type IV secretion system
