# Supplementary material for: Characterization of an NDM-5 carbapenemase-producing Escherichia coli ST156 isolate from a poultry farm in Zhejiang, China
Source: BMC Microbiol. 2019 Apr 25;19:82. doi: 10.1186/s12866-019-1454-2 (PMC6482550; doi:10.1186/s12866-019-1454-2)
Supplement: Supplementary file 1 — Table S1. The gene mutation on the chromosome of strain ECCRA-119. Table S2. Virulence factors of strain ECCRA-119. Table S3. Metal resistance genes of strain ECCRA-119. Figure S1. SNP tree of E. coli ST156 strains. Figure S2. The comparison analysis of 52 blaNDM-5-harboring IncX3 plasmid sequences. Figure S3. Comparison analysis of 24 blaNDM variant sequences based on nucleotide sequences. Figure S4. Phylogenetic relationships between blaNDM variants based on nucleotide sequences. (DOCX 2669 kb) [file 12866_2019_1454_MOESM1_ESM.docx]

Table S1. The gene mutation on the chromosome of strain ECCRA-119

| **Mutation** | **Nucleotide change** | **Amino acid change** | **Resistance** |
| --- | --- | --- | --- |
| *parC* p.S80I | AGC→ATC | S→I | Nalidixic acid,Ciprofloxacin |
| *parE* p.S458A | TCG➝GCG | S➝A | Nalidixic acid,Ciprofloxacin |
| *gyrA* p.S83L | TCG→TTG | S→L | Nalidixic acid,Ciprofloxacin |
| *gyrA* p.D87Y | GAC→TAC | D→Y | Nalidixic acid,Ciprofloxacin |

Table S2. Virulence factors of strain ECCRA-119

| **Virulence factor** | **Location** | **Position in contig** | **Protein function** |
| --- | --- | --- | --- |
| *iss* | Chromosome | 2339233..2339526 | Increased serum survival |
| *gad* | Chromosome | 2435571..2436971 | Glutamate decarboxylase |
| *gad* | Chromosome | 289153..290553 | Glutamate decarboxylase |
| *lpfA* | Chromosome | 4860187..4860759 | Long polar fimbriae |
| *iss* | pTB201 | 102297..102605 | Increased serum survival |
| *iroN* | pTB201 | 114397..116574 | Enterobactin siderophore receptor protein |
| *cma* | pTB201 | 121158..121973 | Colicin M |

Table S3. Metal resistance genes of strain ECCRA-119

| **Gene** | **Location** | **Position in contig** | **Protein function** |
| --- | --- | --- | --- |
| *merA* | pTB201 | 140365..142056 | mercuric reductase |


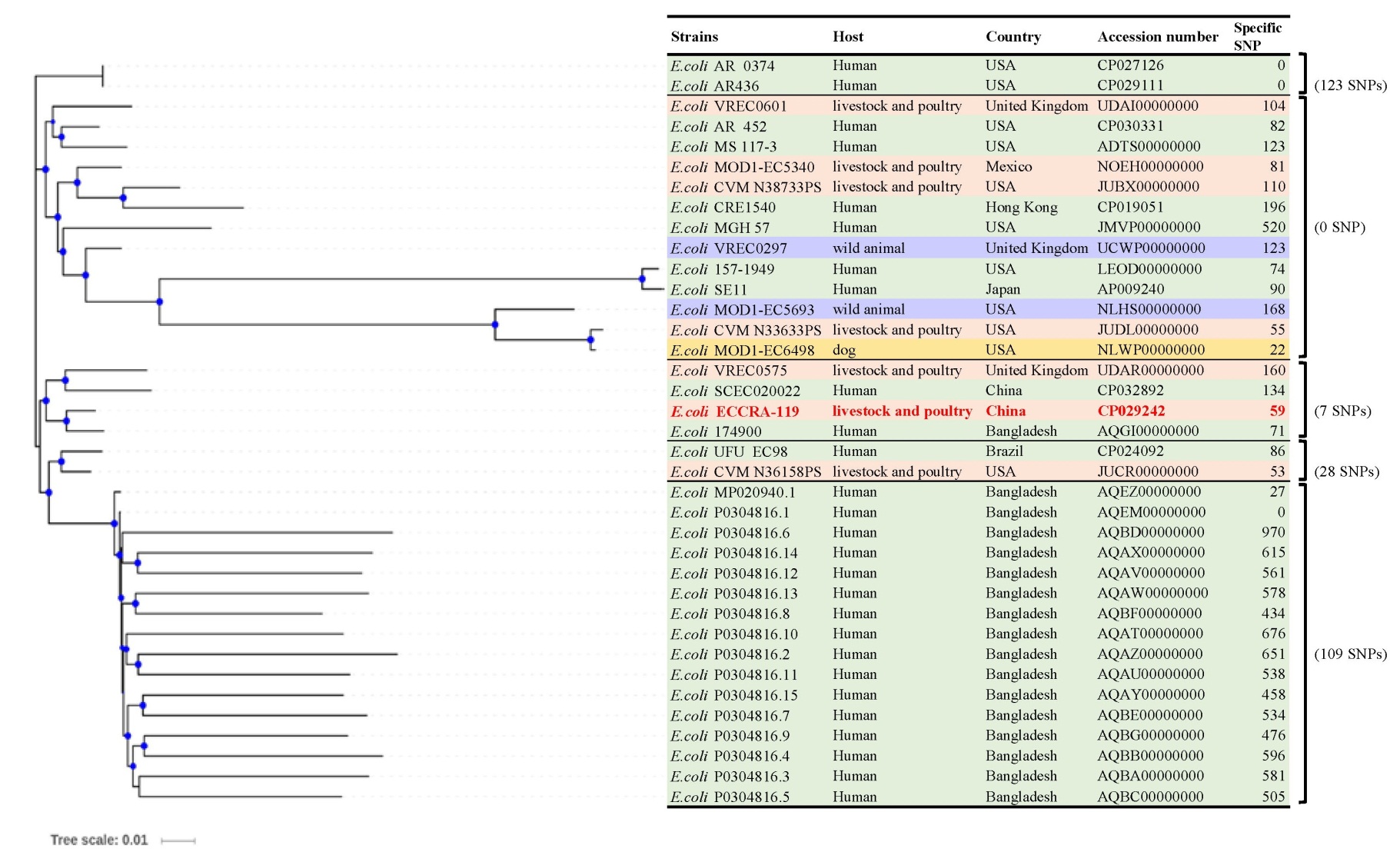


Figure S1. SNP tree of *E.coli* ST156 strains. 37 genome sequences of *E.coli* ST156 strains were conducted phylogenetic analysis based on maximum-likelihood method using the bootstrap resampling method with 100 repeats, showing the position and phylogenetic relationship. Bootstrap values of above 50% are shown in blue points. Strain strain-specific SNPs, isolation source, the isolated country and accession number are provided for each strain. Groups-specific core SNPs are placed in the right brackets. Bar, 0.01 nucleotide substitutions per site.


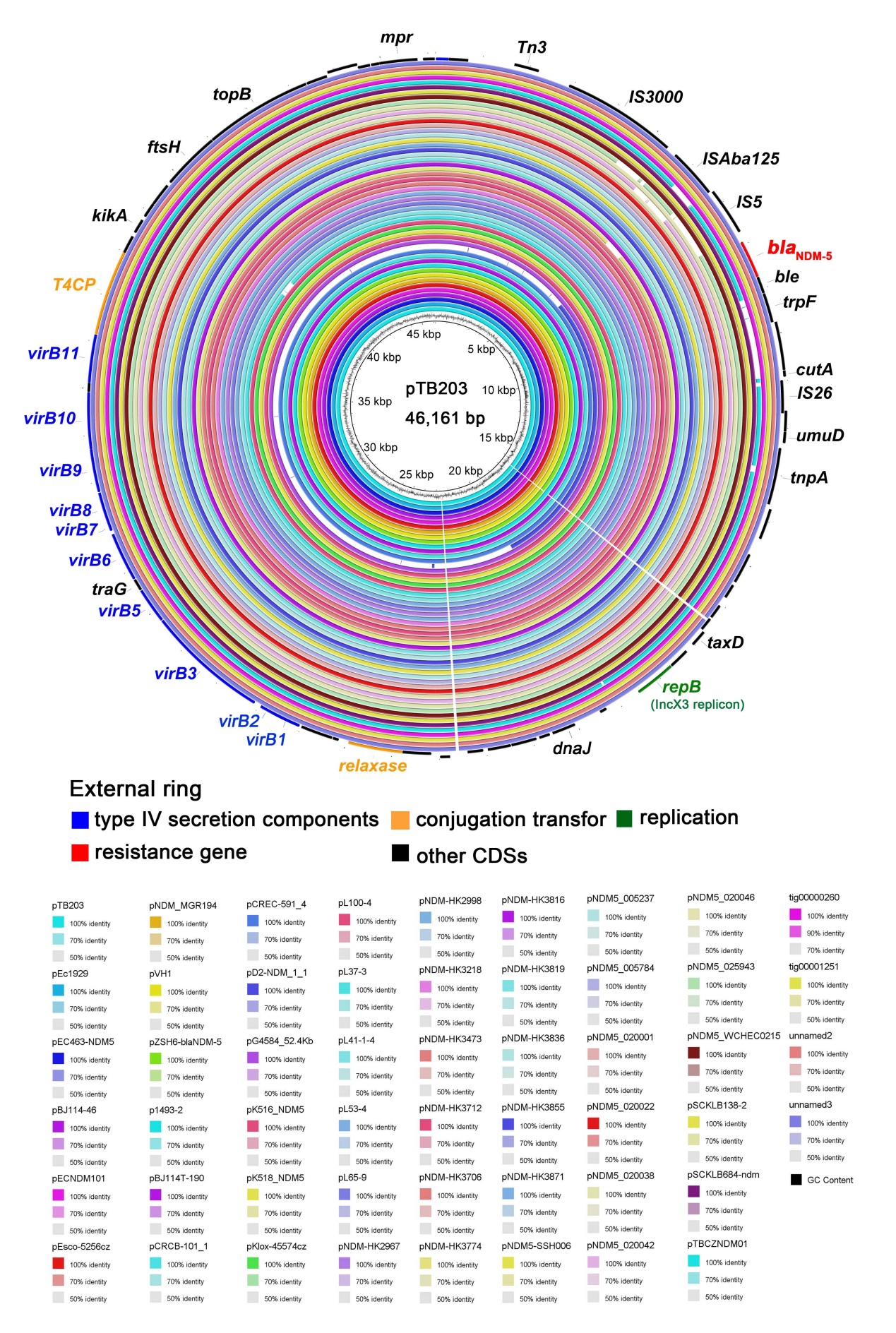


Figure S2. The comparison analysis of 52 *bla*_NDM-5_-harboring plasmid sequences.


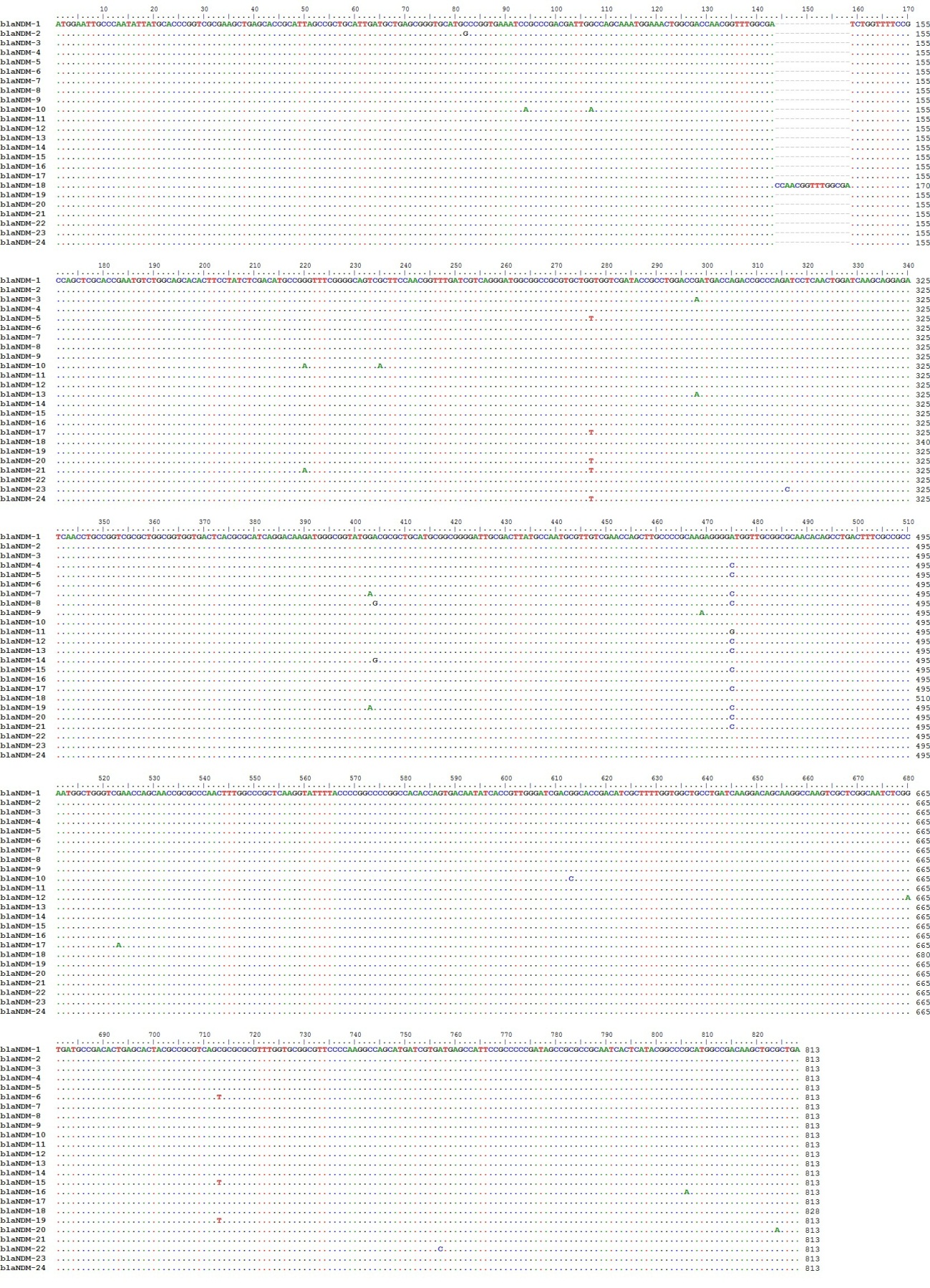


Figure S3. Comparison analysis of 24 *bla*_NDM_ variant sequences based on nucleotide sequences.


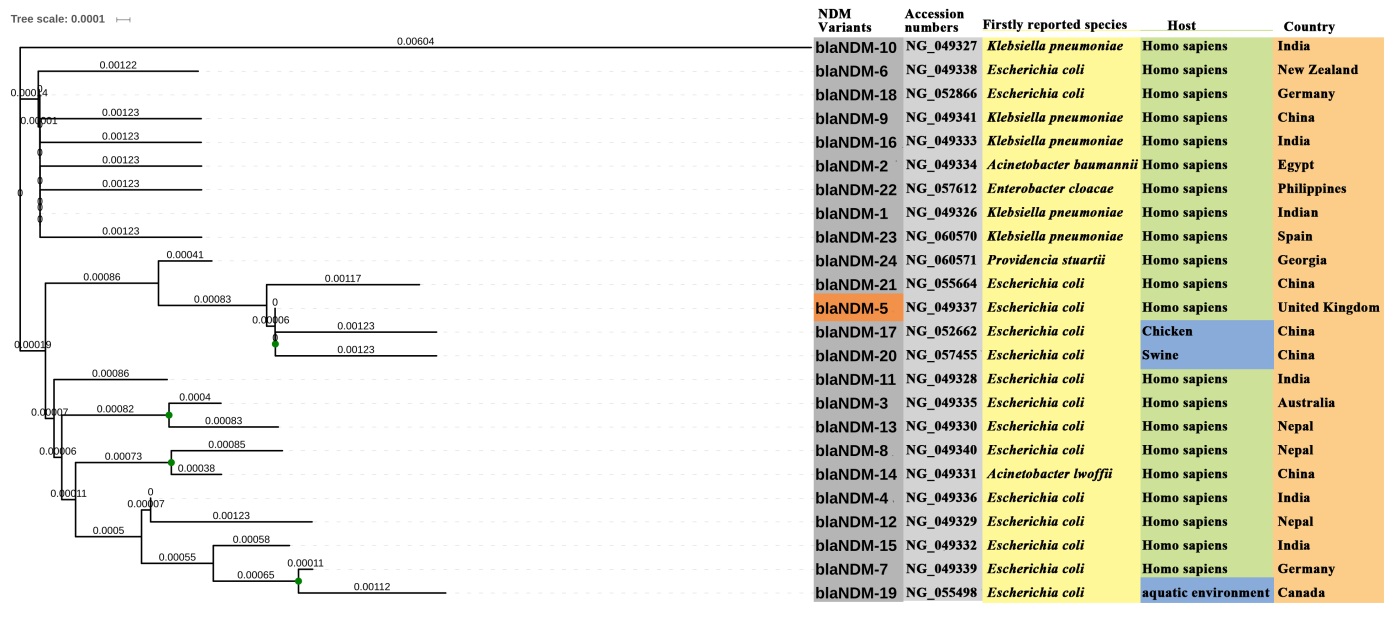


Figure S4. Phylogenetic relationships between *bla*_NDM_ variants based on nucleotide sequences. The tree was generated in MEGA X and iTOL using the neighbour-joining method. The numbers on the branches signify represent branch lengths. Bootstrap values of above 50% are shown in green points.
